# Phosphatidylinositol 4-kinase is a viable target for the radical cure of *Babesia microti* infection in immunocompromised hosts

**DOI:** 10.3389/fcimb.2022.1048962

**Published:** 2022-11-14

**Authors:** Shengwei Ji, Eloiza May Galon, Moaz M. Amer, Iqra Zafar, Masashi Yanagawa, Masahito Asada, Jinlin Zhou, Mingming Liu, Xuenan Xuan

**Affiliations:** ^1^ National Research Center for Protozoan Diseases, Obihiro University of Agriculture and Veterinary Medicine, Obihiro, Hokkaido, Japan; ^2^ Biotechnology Department, Animal Health Research Institute, Dokki, Egypt; ^3^ Department of Veterinary Medicine, Obihiro University of Agriculture and Veterinary Medicine, Obihiro, Hokkaido, Japan; ^4^ Shanghai Veterinary Research Institute, Chinese Academy of Agricultural Sciences, Shanghai, China; ^5^ School of Basic Medicine, Hubei University of Arts and Science, Xiangyang, China

**Keywords:** *Babesia microti*, babesiosis, phosphatidylinositol 4-kinase, treatment, MMV390048

## Abstract

Human babesiosis is a global emerging tick-borne disease caused by infection with intra-erythrocytic parasites of the genus *Babesia*. With the rise in human babesiosis cases, the discovery and development of new anti-*Babesia* drugs are essential. Phosphatidylinositol 4-kinase (PI4K) is a widely present eukaryotic enzyme that phosphorylates lipids to regulate intracellular signaling and trafficking. Previously, we have shown that MMV390048, an inhibitor of PI4K, showed potent inhibition against *Babesia* species, revealing PI4K as a druggable target for babesiosis. However, twice-administered, 7-day regimens failed to clear *Babesia microti* parasites from the immunocompromised host. Hence, in this study, we wanted to clarify whether targeting PI4K has the potential for the radical cure of babesiosis. In a *B. microti*-infected SCID mouse model, a 64-day-consecutive treatment with MMV390048 resulted in the clearance of parasites. Meanwhile, an atovaquone (ATO) resistant parasite line was isolated from the group treated with ATO plus azithromycin. A nonsynonymous variant in the Y272C of the *cytochrome b* gene was confirmed by sequencing. Likewise, MMV390048 showed potent inhibition against ATO-resistant parasites. These results provide evidence of PI4K as a viable drug target for the radical cure of babesiosis, which will contribute to designing new compounds that can eradicate parasites.

## Introduction

Human babesiosis is a tick-borne disease caused by several *Babesia* species, of which *Babesia microti* is one of the main agents (Vannier et al., 2015). *B. microti* infection shows a wide spectrum of symptoms from asymptomatic to fatal disease, particularly in immunocompromised or elderly patients (Vannier et al., 2015). Most cases occur in the United States (U.S.), especially in the northeastern and northern midwestern regions (Krause, 2019). For the time being, the treatment of human babesiosis, as recommended by Centers for Disease Control and Prevention (CDC), usually involves the combination of atovaquone (ATO) plus azithromycin (AZI) for 1-2 weeks, which exhibits fewer side effects compared to an alternative combined therapy of clindamycin and quinine (Vannier and Krause, 2012). The treatment period for babesiosis may extend to six weeks or more in severely immunocompromised patients, and *cytochrome b* (*Cytb*) and *ribosomal protein subunit L4* (*rpl4*) mutations were associated with parasite resistance to ATO and AZI, respectively, resulting in treatment failure (Krause et al., 2008; Wormser et al., 2010; Vannier and Krause, 2012; Simon et al., 2017). Another recommended regimen for babesiosis, clindamycin combined with quinine, is frequently associated with side effects, such as hearing loss, vertigo, and tinnitus (Renard and Ben Mamoun, 2021). Due to limited options, this treatment is still recommended for patients with severe babesiosis (Renard and Ben Mamoun, 2021). Moreover, previous reports revealed that monotherapy with quinine, clindamycin, or AZI was ineffective against *B. microti* infection in immunocompromised hosts (Lawres et al., 2016). Therefore, the buildout of new drug candidates or targets is urgently needed for the control and treatment of human babesiosis. Phosphatidylinositol 4-kinase (PI4K) is a ubiquitous eukaryotic lipid kinase involved in the production of phosphatidylinositol 4-phosphate (PI4P) (Li et al., 2021). PI4K has been reported to play a key role in the occurrence and development of cancer, viral infections, and malaria (Li et al., 2021). PI4K not only exhibits the potential for eliminating malaria (McNamara et al., 2013; Paquet et al., 2017), but also possesses an inhibitory effect against *Babesia* species, as we recently reported (Ji et al., 2022). The 2-aminopyridine MMV390048, a representative of a new chemical class of *Plasmodium* PI4K inhibitor (Paquet et al., 2017), showed potent inhibition against *B. gibsoni in vitro*, and against *B. rodhaini* and *B. microti in vivo* (Ji et al., 2022). However, twice-administered, short-time treatment did not eliminate the parasite from immunocompromised hosts. Hence, the purpose of this study was to test whether uninterrupted treatment targeting *B. microti* PI4K could eradicate *B. microti* infection in immunocompromised hosts.

## Materials and methods

### Chemical reagents

MMV390048, sesame oil (SO), atovaquone (ATO), and azithromycin (AZI) were purchased from Sigma-Aldrich (Tokyo, Japan). MMV390048, ATO, and AZI were dissolved in SO to prepare a stock solution with a concentration of 40 mg/ml and kept at 4°C before use. KOD FX Neo DNA polymerase was purchased from Toyobo (Tokyo, Japan). The Big Dye Terminator v3.1 cycle sequencing kit was purchased from Applied Biosystems (Tokyo, Japan).

### Parasites and mice

*Babesia microti* Peabody mjr strain (ATCC^®^ PRA-99™) was purchased from ATCC and stocked in our laboratory. For the maintenance of *B. microti*, cryopreserved parasitized RBCs were passaged by intraperitoneal (i.p.) injection in donor mice. Six-week-old female severe combined immunodeficiency (SCID) mice and BALB/c mice were purchased from CLEA Japan (Tokyo, Japan) and used for *in vivo* studies.

### Mouse infection and drug administration

To confirm the efficacy of uninterrupted treatment targeting PI4K, 15 SCID mice were randomly divided equally into 3 groups and intraperitoneally injected with 1 × 10^7^ *B. microti*. Blood smears were prepared every other day, and the hematocrit (HCT) was measured every four days. Treatment was initiated at 4 days post-infection (DPI) when mouse parasitemia is ~1%. This timeline was followed since early clinical manifestations are observed when parasitemia exceeds 1% in babesiosis patients (Akel and Mobarakai, 2017). Daily treatment with 20 mg/kg MMV390048, 20 mg/kg ATO plus 20 mg/kg AZI, and 0.2 ml vehicle (sesame oil) was given orally to each group, respectively (Batiha et al., 2020; Tuvshintulga et al., 2022). These treatments were discontinued when mice tested negative by PCR detection of *B. microti* 18S ribosomal RNA (18S-rRNA) gene. To isolate the *B. microti Cytb* mutant strain, parasites from the SCID mouse treated with ATO plus AZI were collected and passaged in a donor SCID mouse. To evaluate the efficacy of PI4K inhibitor on ATO-resistant parasites, 15 BALB/c mice were randomly divided equally into 3 groups and intraperitoneally injected with 1 × 10^7^ *B. microti Cytb* mutant strain. A 7-day treatment was given to mouse groups as described above and the parasitemia and HCT levels were monitored.

### Detection of *B. microti* 18S-rRNA gene and surveillance of gene variants

Blood samples were collected from the tail vein and were diluted in PBS, followed by incubation at 100°C for 5 min. After incubation, the samples were centrifuged at 10,000 rpm for 5 min and the supernatants were collected and used for detection. To rule out false negative results, the samples were checked using Qubit™ 1 × dsDNA BR assay kit (Thermo Fisher Scientific) and Qubit^®^ 2.0 fluorometer (Thermo Fisher Scientific) before running the PCR assay to ensure that genomic DNA was present. Detection of the 18S-rRNA gene started 16 DPI and was used to evaluate whether the parasites were cleared from the SCID mice. Gene amplification was performed following a previously described protocol (Persing et al., 1992). The *Cytb* and *rpl4* mutations were determined by Sanger sequencing (Tuvshintulga et al., 2022). The obtained sequence was aligned with the wild type sequence. A genetic variant was detected in *Cytb* gene and deposited in GenBank database with accession no. ON815034.

### Statistical analysis

The differences in parasitemia and HCT between control and treated groups were analyzed using the one-way analysis of variance using GraphPad Prism (La Jolla, CA, USA). A *P* value of < 0.05 was considered to be statistically significant.

## Results

### Radical cure of human babesiosis by uninterrupted treatment targeting phosphatidylinositol 4-kinase

**Figure 1:**
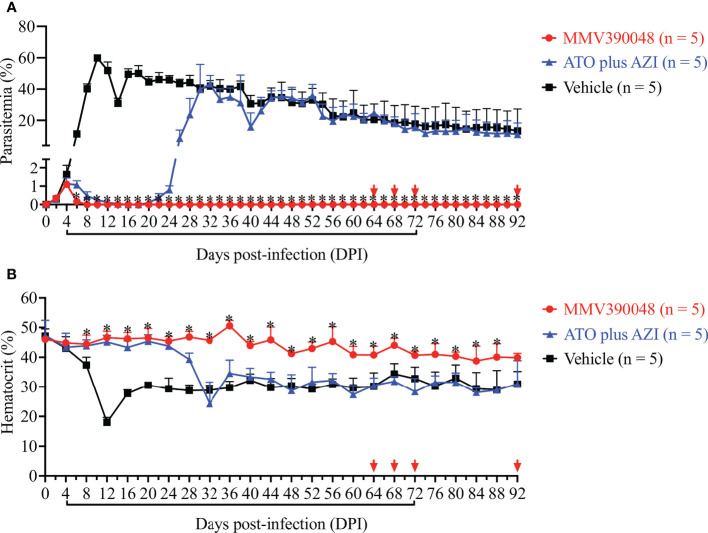
The efficacy of consecutive treatment with MMV390048 in *Babesia microti*-infected severe combined immunodeficiency (SCID) mice. **(A)** Course of parasitemia in vehicle-, or atovaquone (ATO) plus azithromycin- (AZI), or MMV390048-treated groups. **(B)** Hematocrit changes in *B. microti*-infected SCID mice treated with vehicle, or ATO plus AZI, or MMV390048. Treatment was given orally starting from 4 days post-infection (DPI). The black lines indicate time of treatment and the red arrows indicate testing negative in PCR assay. The asterisks indicate a significant difference (*P* < 0.05) between the drug-treated groups and vehicle-treated group. Parasitemia was calculated by counting infected RBCs among 3,000 RBCs using Giemsa-stained blood smears.

**Figure 2:**
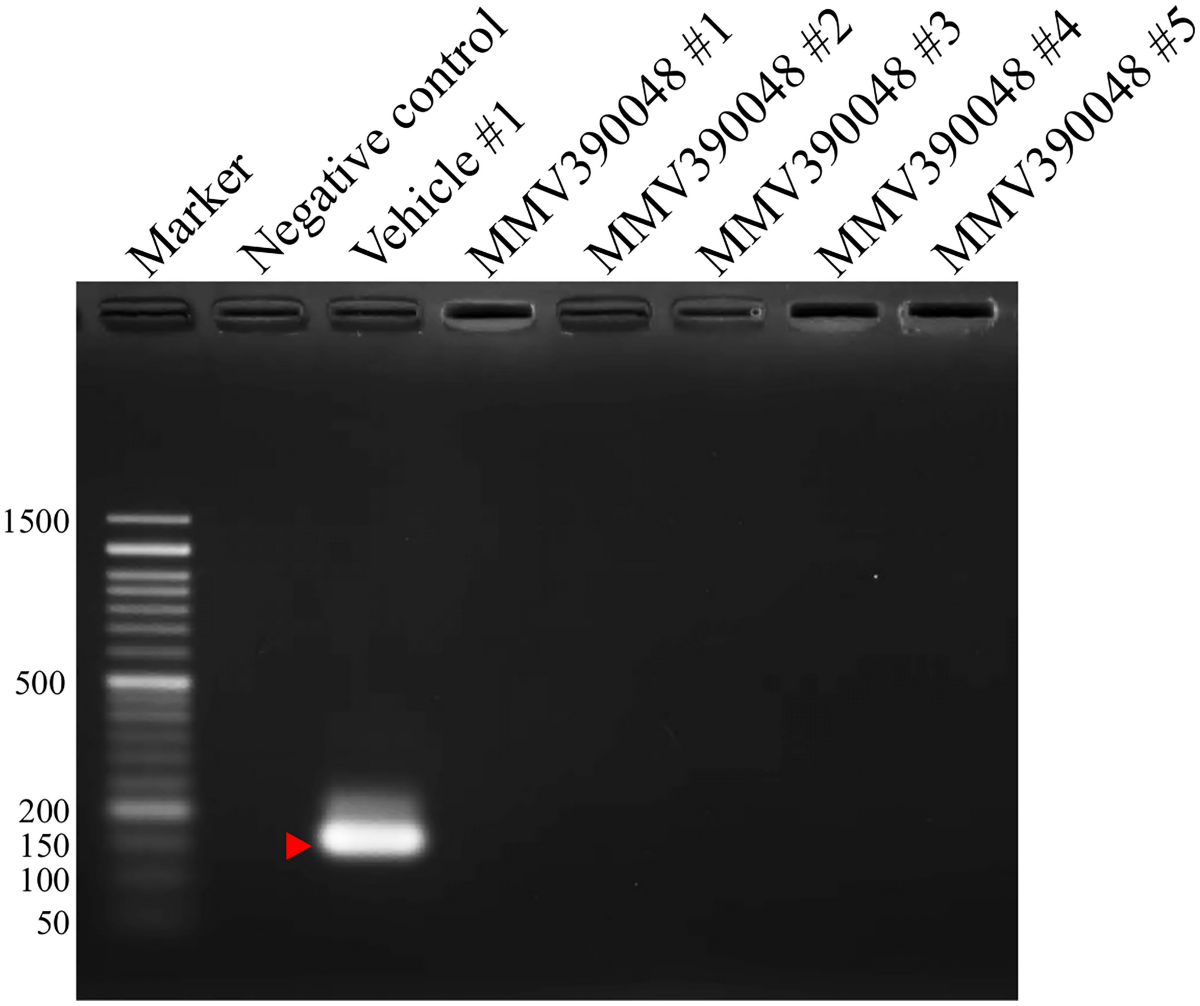
Molecular detection of parasite DNA in the blood of MMV390048-treated group (#1-5) at 92 DPI. The red arrow shows the expected band length of 154 bp for the *B. microti* 18S-rRNA gene.

**Figure 3:**
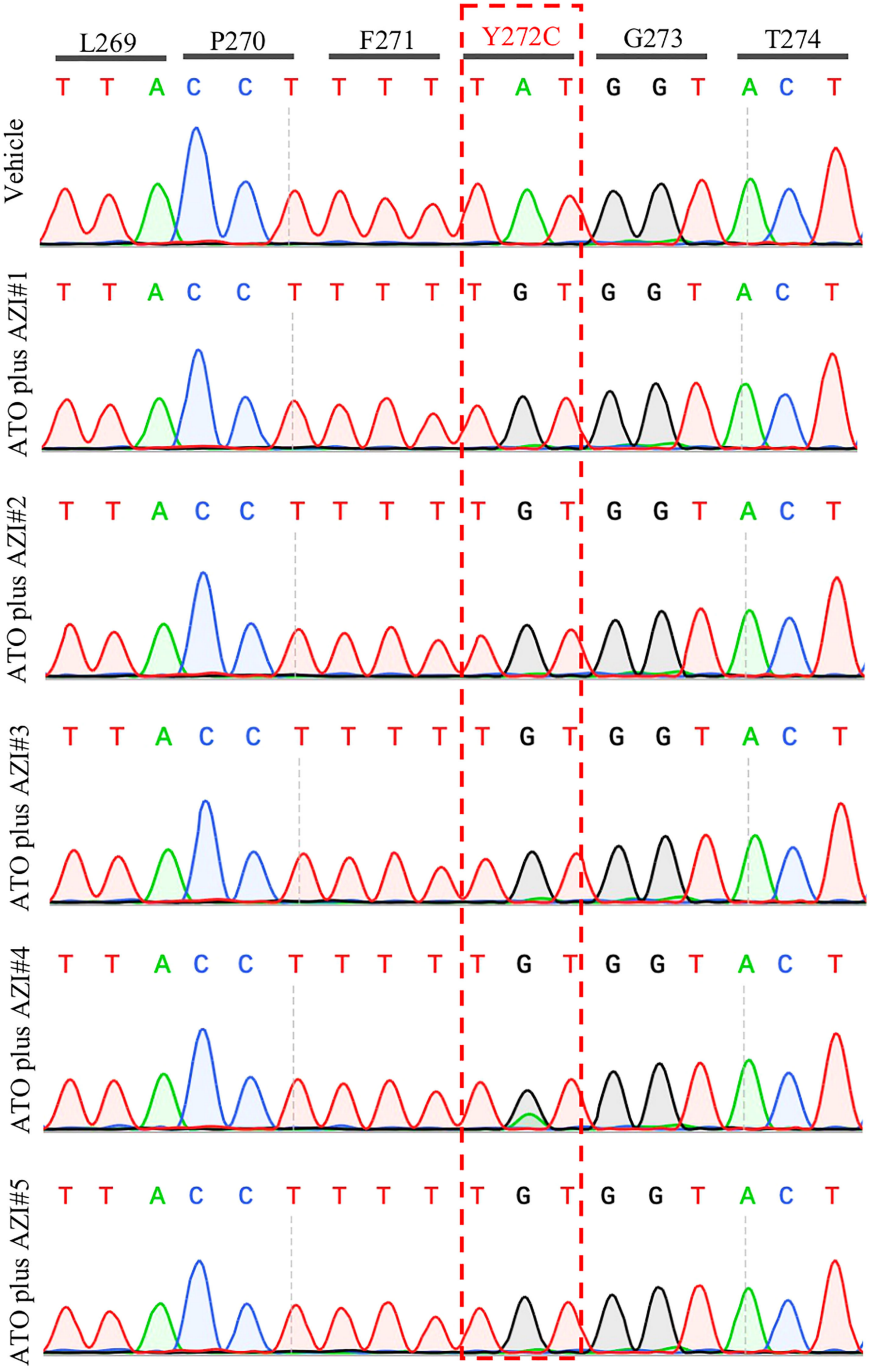
Representative sequencing chromatogram of *cytochrome b* (*Cytb*) gene in recrudescent parasites. The parasite DNA of vehicle #1 and ATO plus AZI (#1-5) were extracted from a blood sample at 92 days post-infection and was used to amplify and sequence the *B. microti Cytb* gene.

Treatment with MMV390048 showed potent efficacy against *B. microti*, evidenced by abated parasitemia from 5 DPI and undetectable parasites in blood smears from 8 DPI (**Figure 1A**). Moreover, parasites were no longer detectable by PCR from 64 DPI to 92 DPI (**Figure 2**). At 10 DPI, parasites in the vehicle-treated group reached the highest parasitemia (average 60.6%), with a transient and slight decline at 14 DPI, and maintained fluctuating parasitemia until the end of the trial. The mean parasitemia significantly differed between the vehicle-treated and MMV390048-treated groups from 6 DPI (**Figure 1A**). In ATO plus AZI-treated group, parasites were initially inhibited until 22 DPI, but the parasitemia rapidly increased from 24 DPI and reached its peak at 32 DPI (average 40.1%) (**Figure 1A**). From 30 DPI, the ATO plus AZI was ineffective on parasite growth as no significant difference in parasitemia was observed when compared to the vehicle-treated group. In addition, markedly lower HCT levels were recorded in vehicle-treated and ATO plus AZI-treated groups from 8 DPI and 32 DPI, respectively (**Figure 1B**). The *Cytb* and *rpl4* genes were sequenced from relapsed parasites and a single nucleotide variant (SNV) of the *Cytb* gene was detected as a non-synonymous coding change at position 272 (Y272C) (**Figure 3**). Meanwhile, there was no mutation detected in the *rpl4* gene.

### Inhibitory efficacy of MMV390048 against ATO-resistant *B. microti* strain

**Figure 4:**
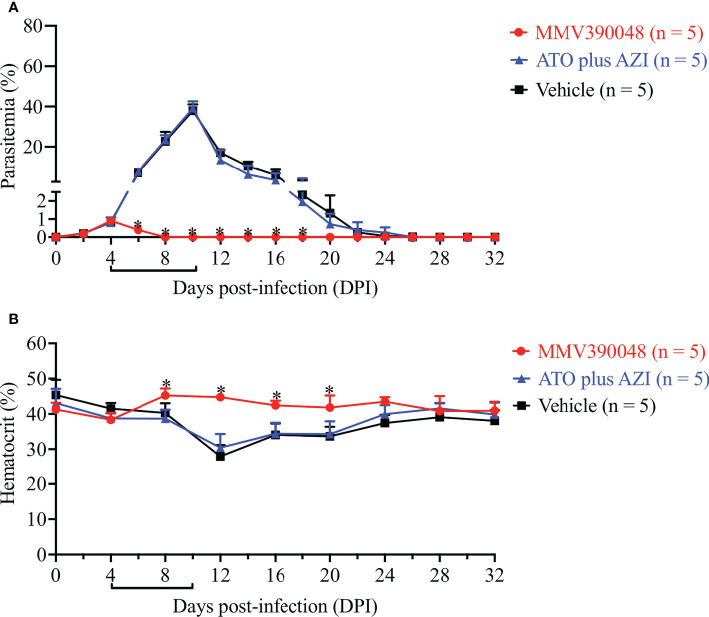
The efficacy of MMV390048 against *B. microti Cytb* mutant strain in BALB/c mice. **(A)** Course of parasitemia of vehicle- or ATO plus AZI-, or MMV390048-treated BALB/c mouse groups. **(B)** Hematocrit changes of *B. microti Cytb* mutant strain in mice treated with vehicle, or ATO plus AZI, or MMV390048. The black lines indicate the time of treatment. The asterisks indicate a significant difference (*P* < 0.05) between the drug-treated groups and vehicle-treated group. Parasitemia was calculated by counting infected RBCs among 3,000 RBCs using Giemsa-stained blood smears.

The next step was to evaluate the sensitivity of *B. microti Cytb* mutant strain to MMV390048. In the vehicle-treated group, *B. microti Cytb* mutant strain rapidly increased in mice and reached peak parasitemia at 10 DPI (average 36.7%) and a lower HCT level was observed from 12 DPI (**Figure 4**). As expected, ATO plus AZI was ineffective against the *B. microti Cytb* mutant strain (**Figure 4A**). No significant difference in the level of parasitemia was observed between vehicle- and ATO plus AZI-treated groups. In contrast, the growth of *B. microti Cytb* mutant strain and the development of anemia were significantly inhibited upon treating mice with MMV390048 (**Figure 4B**).

## Discussion

To avoid developing drug resistance, the treatment for human babesiosis usually consists of a two-drug combination, such as ATO plus AZI (Krause et al., 2000). Despite this, acquired drug resistance is well documented in some severe cases in immunocompromised patients (Krause et al., 2008; Wormser et al., 2010; Vannier and Krause, 2012; Simon et al., 2017). Hence, the radical cure of babesiosis remains challenging in severely immunocompromised patients. In the recent past, a few compounds have been reported as promising drugs against human babesiosis, namely endochin-like quinolones (ELQs) (Lawres et al., 2016; Chiu et al., 2021), tafenoquine (Mordue and Wormser, 2019; Liu et al., 2021), and clofazimine (Tuvshintulga et al., 2022). ELQs showed inhibitory effects against apicomplexan parasites by targeting *Cytb* (Doggett et al., 2012; Stickles et al., 2015). In babesiosis, a 7-day treatment of ELQ-334 plus ATO prevented the recrudescence in the SCID mouse model of *B. microti* infection (Lawres et al., 2016). Similarly, a 10-day treatment of ELQ-502 monotherapy or in combination with ATO resulted in the radical cure of babesiosis with no recrudescence in the mouse model (Chiu et al., 2021). Tafenoquine was approved by U. S. Food and Drug Administration (FDA) in 2018 for the radical cure of *Plasmodium vivax* infection and chemoprophylaxis of malaria (Watson et al., 2021). In SCID mice, tafenoquine showed strong inhibition against *B. microti* infection, evident from the single dose requirement (Mordue and Wormser, 2019; Liu et al., 2021). Tafenoquine treatment in cases of relapsing babesiosis caused by drug-resistant *B. microti* is followed by resolution of parasitemia and symptoms in the patient, demonstrating tafenoquine’s excellent effectivity in clinical settings (Marcos et al., 2022; Rogers et al., 2022). However, the use of tafenoquine entails the risk of inducing severe hemolytic anemia in G6PD-deficient patients (Peters and Van Noorden, 2009). Clofazimine combined with ATO was also evaluated as a candidate for human babesiosis. Uninterrupted treatment of clofazimine with ATO resulted in the radical cure of *B. microti*-infected SCID mice in 44 days (Tuvshintulga et al., 2022). Phosphatidylinositol kinases (PIKs) are essential in the regulation of cell proliferation, survival, and membrane trafficking (Hassett and Roepe, 2018). Currently, six *P. falciparum* genes are hypothesized to encode PIKs, while in *Babesia* species, these genes are still unidentified (Hassett and Roepe, 2018). In *Plasmodium*, PI4K phosphorylates lipids to regulate intracellular signaling and trafficking, making it a druggable target to eliminate malaria (McNamara et al., 2013). In blood stages, the inhibitor prevents the parasite’s development by disrupting plasma membrane ingression around the developing daughter merozoites. *B. microti* PI4K shares an identity value of 62.8% with *P. falciparum* and is highly conserved among *Babesia* species (Ji et al., 2022). Hence, we speculate that the mechanism in *Plasmodium* also applies to *Babesia* species. MMV390048 is an inhibitor of *Plasmodium* PI4K which was under evaluation in human clinical trials (Sinxadi et al., 2020). A previous study reported MMV390048 has potent inhibition against *Babesia* species by targeting PI4K, revealing a promising druggable target (Ji et al., 2022). In light of this, we further examined if targeting PI4K by monotherapy is sufficient to achieve the radical cure of *B. microti* infection in SCID mice. In this study, we used MMV390048 as an inhibitor for *B. microti* PI4K (*Bm*PI4K) and a 64-day uninterrupted treatment with MMV390048 succeeded in curing babesiosis in *B. microti*-infected SCID mice. Although this therapy was longer than in ELQ-502 (10 days) and clofazimine with ATO (44 days), we have confirmed that PI4K is a promising target for the clearance of parasites. Meanwhile, in ATO plus AZI-treatment group, the parasite established resistance to ATO and AZI, in addition to the development of a single amino acid mutation (Y272C) in the *B. microti Cytb* gene. Y272 is highly conserved among apicomplexan parasites and the site mutation of Y272 will confer a high degree of resistance to the drug (Fisher and Meunier, 2008; Lemieux et al., 2016). Moreover, *B. microti* Y272C mutation has been described in a clinical case and caused treatment failure (Simon et al., 2017). MMV390048 also demonstrated potent inhibition of *B. microti Cytb* mutant strain. Despite MMV390048 exhibiting a good inhibitory effect, *Babesia* tends to be more tolerant than *Plasmodium* when treated with MMV390048. Hence, if MMV390048 were to be used for treating human babesiosis, the recommended dose should be higher than 120 mg (a safety dose test in human clinical trials) (Sinxadi et al., 2020; Ji et al., 2022). Moreover, the accompanying safety issues should be considered and future work can focus on developing and designing *Babesia* PI4K-specific inhibitors, as well as the development of MMV390048-combined therapeutics. This will accelerate the development of next-generation anti-babesiosis therapeutics to eliminate *Babesia* infection. In summary, results from the present study revealed that targeting *Bm*PI4K not only suppresses parasite growth but also eradicates parasites in immunocompromised hosts, especially in relapsing infections caused by ATO-resistant *B. microti* strain.

## Conclusion

Based on the preceding findings, we conclude that *Bm*PI4K is a feasible and promising drug target for the elimination of *B. microti* infection. This study opens new avenues to rationally design inhibitors with improved drug-like properties against *Babesia* species.

## Data availability statement

The datasets presented in this study can be found in online repositories. The names of the repository/repositories and accession number(s) can be found below: https://www.ncbi.nlm.nih.gov/nuccore/ON815034.

## Ethics statement

The animal study was reviewed and approved by the Research Ethics Review Committee of the Obihiro University of Agriculture and Veterinary Medicine.

## Author contributions

SJ, ML, and XX designed the study. SJ carried out the experiments. IZ contributed reagents/materials preparation. SJ, EG, MMA, MY, MA, and JZ wrote the manuscript. All authors read and approved the final manuscript.

## Funding

This project was funded by a Grant-in-Aid for Scientific Research (18H02336 and 18KK0188) and the Japan Society for the Promotion of Science Core-to-Core program, (both grants are from the Ministry of Education, Culture, Sports, Science and Technology of Japan), in addition to a grant from Strategic International Collaborative Research Project (JPJ008837) promoted by the Ministry of Agriculture, Forestry and Fisheries of Japan. This research was also partially funded by the Central Public-interest Scientific Institution Basal Research Fund of China (Y2021GH01-3).

## Conflict of interest

The authors declare that the research was conducted in the absence of any commercial or financial relationships that could be construed as a potential conflict of interest.

## Publisher’s note

All claims expressed in this article are solely those of the authors and do not necessarily represent those of their affiliated organizations, or those of the publisher, the editors and the reviewers. Any product that may be evaluated in this article, or claim that may be made by its manufacturer, is not guaranteed or endorsed by the publisher.
